# Supplementary material for: Rapid neo-sex chromosome evolution and incipient speciation in a major forest pest
Source: Nat Commun. 2017 Nov 17;8:1593. doi: 10.1038/s41467-017-01761-4 (PMC5693900; doi:10.1038/s41467-017-01761-4)
Supplement: Supplementary file 1 — Supplementary Information [file 41467_2017_1761_MOESM1_ESM.pdf]

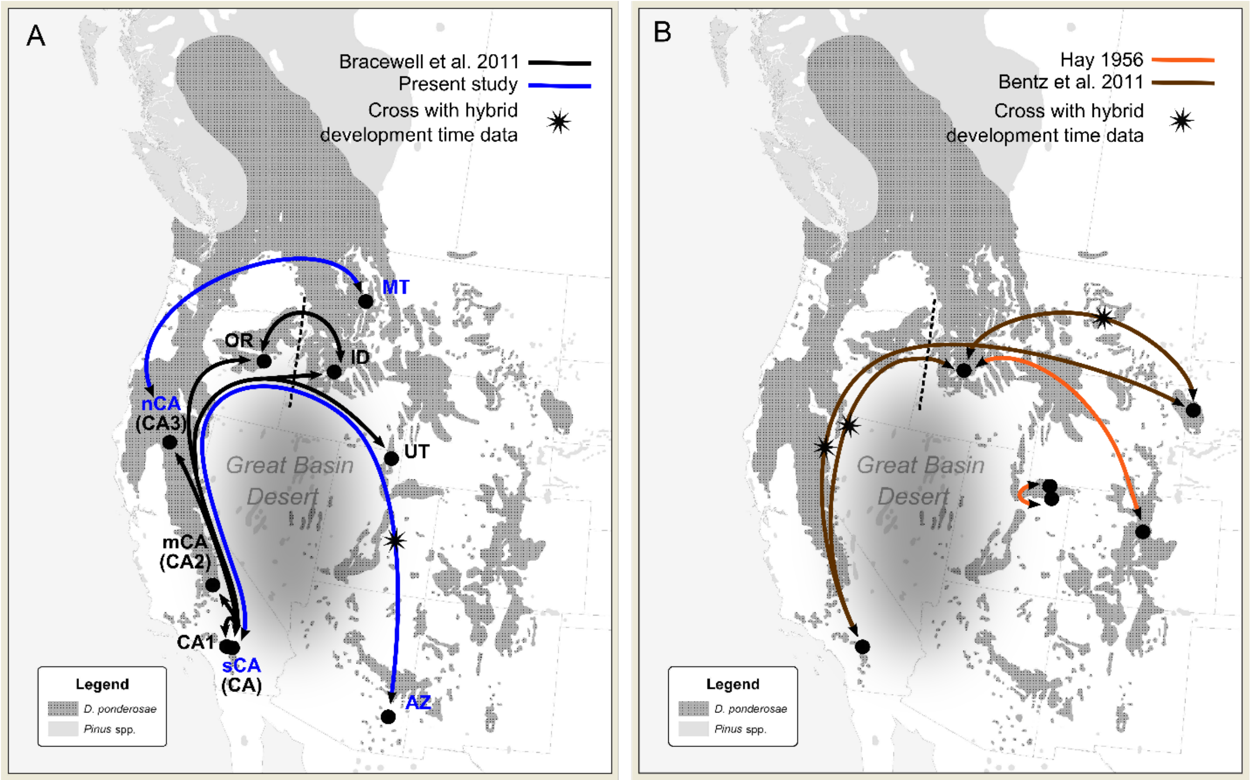

Supplementary Figure 1. Summary of mountain pine beetle crosses. (**A**) Mountain pine beetle population crosses (9 populations, 18 cross-types, 1,109 crosses) from a previous investigation[1] shown with crosses from the present study. Names in parentheses denote population names used in Bracewell *et al.*[1]. (**B**) Two other studies have also crossed mountain pine beetle populations in a manner where hybrid male and female fertility could also be assessed to some extent (Hay[2] and Bentz *et al.*[3]). In total, these four studies are consistent with a central HMS boundary (dotted vertical line) in the mountain pine beetle. Crosses between populations east or west of the HMS boundary do not show HMS. Development time of hybrids, which has been studied less intensely, has been recorded for a subset of populations (shown).

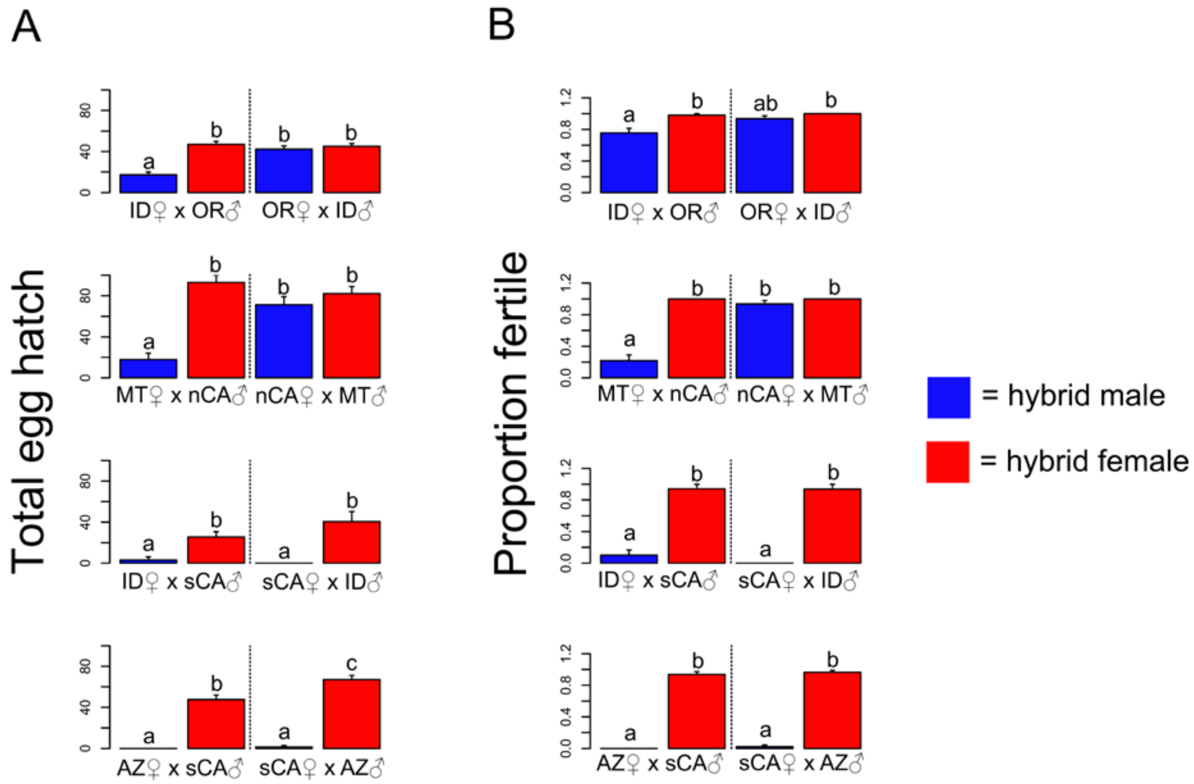

Supplementary Figure 2. Fertility of beetle hybrids. The (**A**) total egg hatch and (**B**) proportion of fertile individuals in crosses between four populations. OR × ID and sCA × ID results are from Bracewell *et al*.[1]. Reciprocal crosses are separated by a dashed line. Total egg hatch summarized from Supplementary Table 1 and Bracewell et al.[1]. For proportion fertile, individuals were considered fertile if ≥ 1 egg hatched. Bars with the same letter are not significantly different within a particular population cross (Tukeys HSD test for total egg hatch and Bonferroni-corrected pairwise proportion test for fertility).

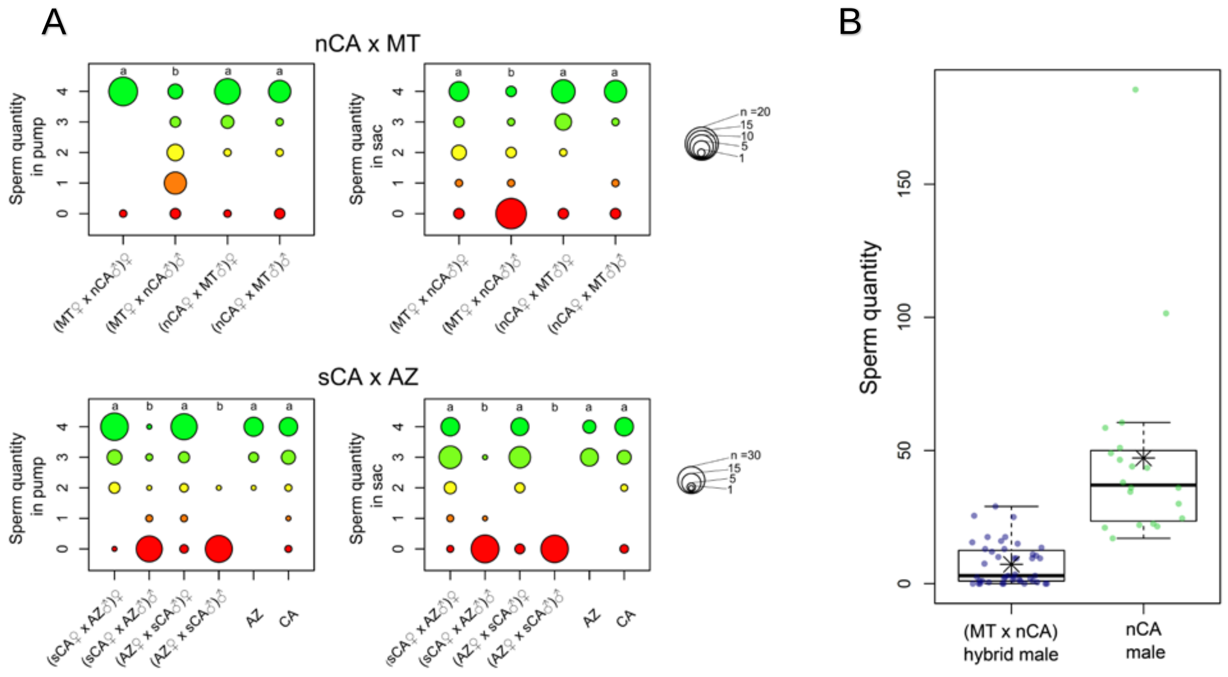

Supplementary Figure 3. Sperm quantity in the reproductive tract of females in crossing experiments and in the seminal vesicles of hybrid males. (**A**) Shown is the quantity of sperm in the spermathecal sac and pump of females from the nCA × MT cross experiment (above) and sCA × AZ cross experiment (below). See Methods for how sperm quantity was determined for male and female reproductive structures. Circle size is proportional to the number of females observed in a particular sperm quantity category (scale shown on the right for each cross experiment). A color gradient was applied to help highlight when the structure was full (score = 4 = green) or when it was empty (score = 0 = red). The population a hybrid was backcrossed to was not found to influence sperm quantity and therefore those results were combined. Crosses that significantly differ in sperm quantity are denoted with different letters (Bonferroni-corrected Wilcoxon rank sum tests of all pairwise comparisons). (**B**) Sperm quantity in the male seminal vesicle of MT♀ × nCA♂ hybrid males and pure population nCA males. Asterisk denotes the mean. Pure population males (nCA) had significantly more sperm in the seminal vesicle than hybrid males (MT × nCA) (Wilcoxon rank sum test; $p < 0.0001$).

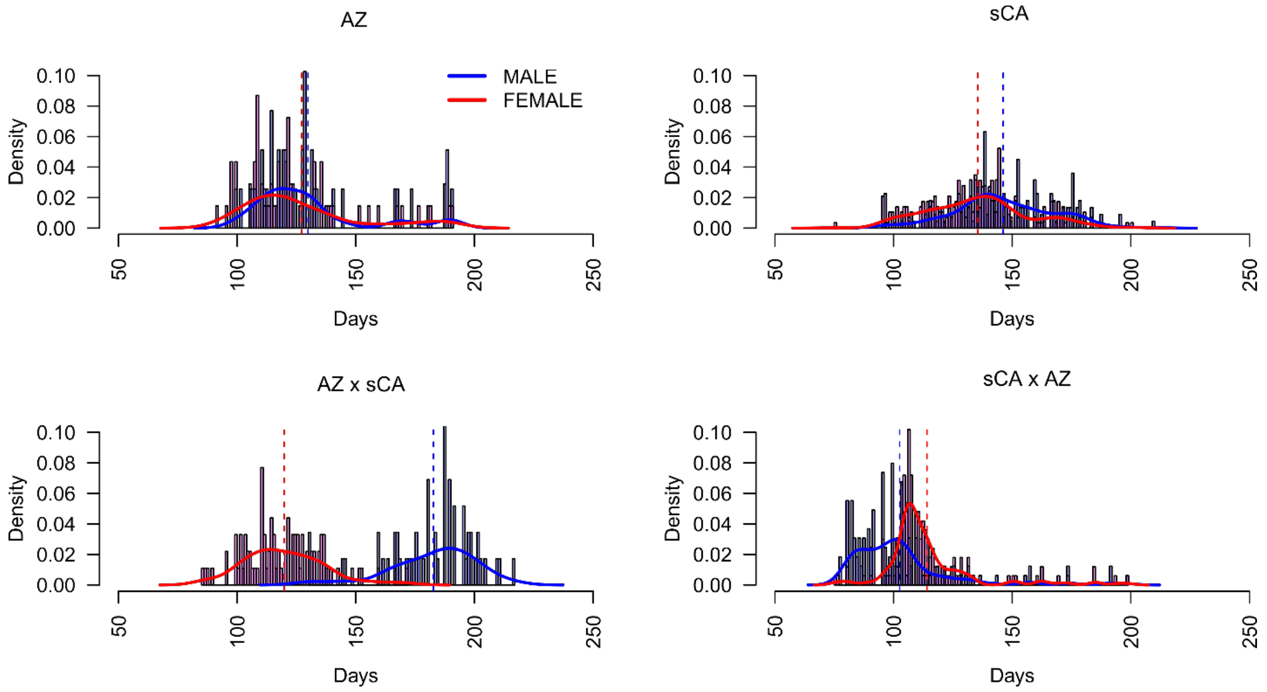

Supplementary Figure 4. Development time of AZ and sCA populations and their reciprocal hybrids. Dashed lines represent the median development time for each sex. Crosses between populations are designated female first. AZ males and females did not differ in emergence time. sCA males emerged slightly, yet significantly later than sCA females (Kruskal-Wallis rank sum test; $p < 0.0001$). AZ × sCA hybrid males showed highly delayed development and emerged far later than hybrid females (Kruskal-Wallis rank sum test; $p < 0.0001$). In contrast, sCA × AZ hybrid males emerged slightly, yet significantly earlier than hybrid females (Kruskal-Wallis rank sum test; $p < 0.0001$).

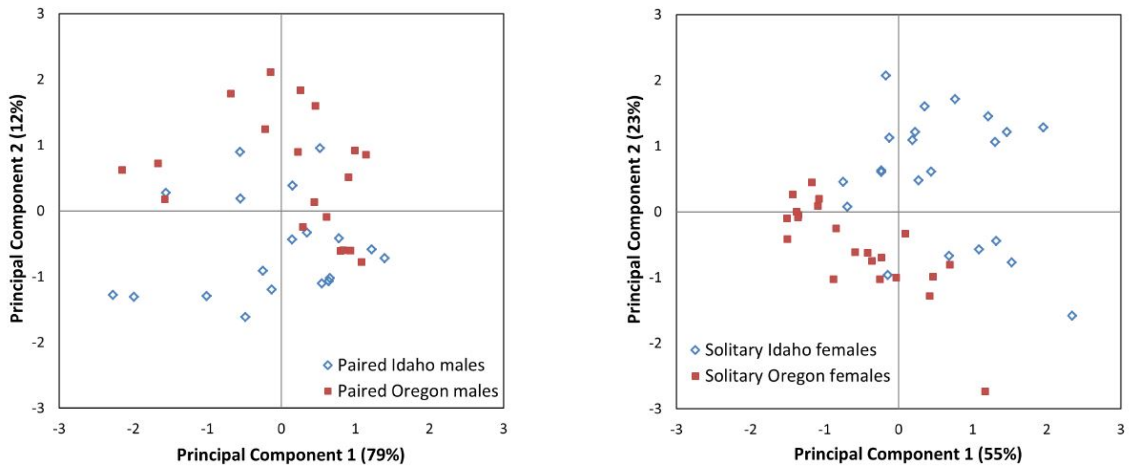

Supplementary Figure 5. Principal component analyses of pheromone profiles of OR and ID mountain pine beetles. Results of GC-MS of pheromone component production for OR (red square) and ID (blue diamond) males paired with females (left panel) and solitary females (right panel).

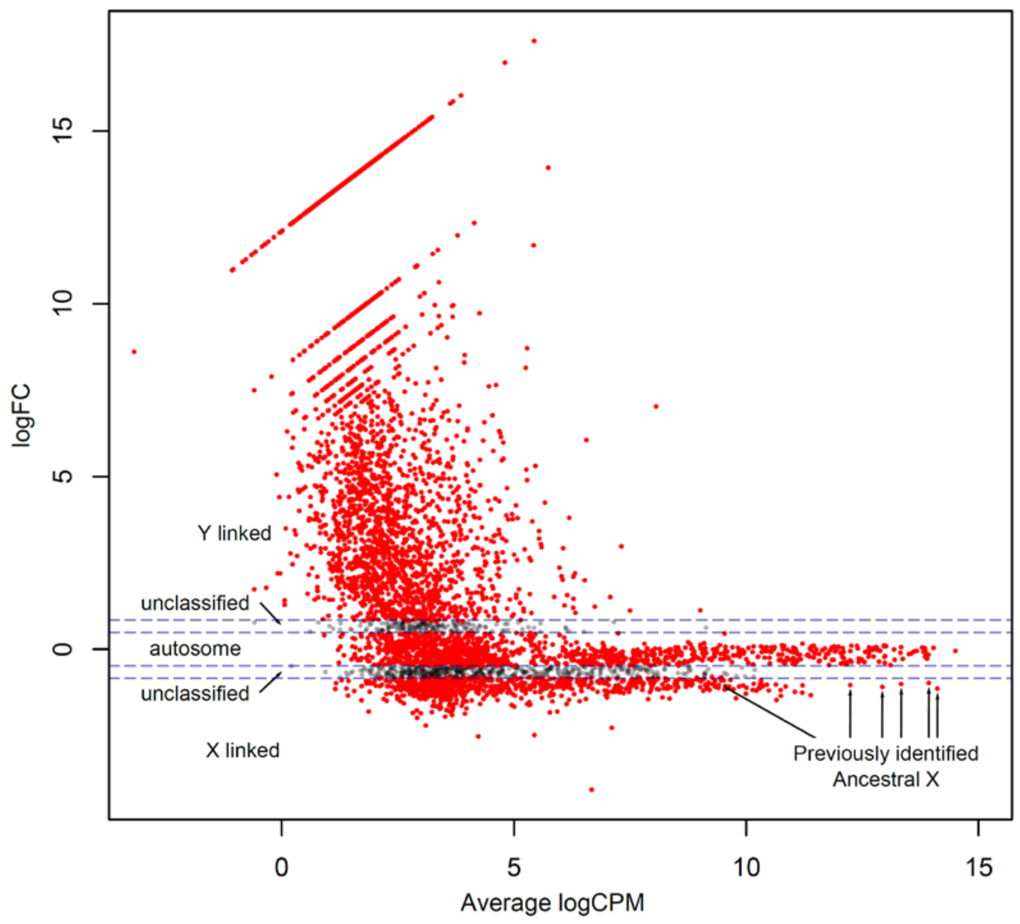

Supplementary Figure 6. Assigning scaffolds to linkage categories. Differences in male and female sequencing coverage (Log_2 fold change, y-axis) over the 4,877 mountain pine beetle male genome scaffolds (Average log Counts Per Million mapped reads, x-axis). Each point represents a different scaffold. Dashed lines denote the cutoffs for the different linkage categories. Previously identified ancestral X chromosome scaffolds are highlighted. For simplicity, values reported in the main text as cutoffs for the different partitions are presented as the absolute value of Log_2 fold change from above.

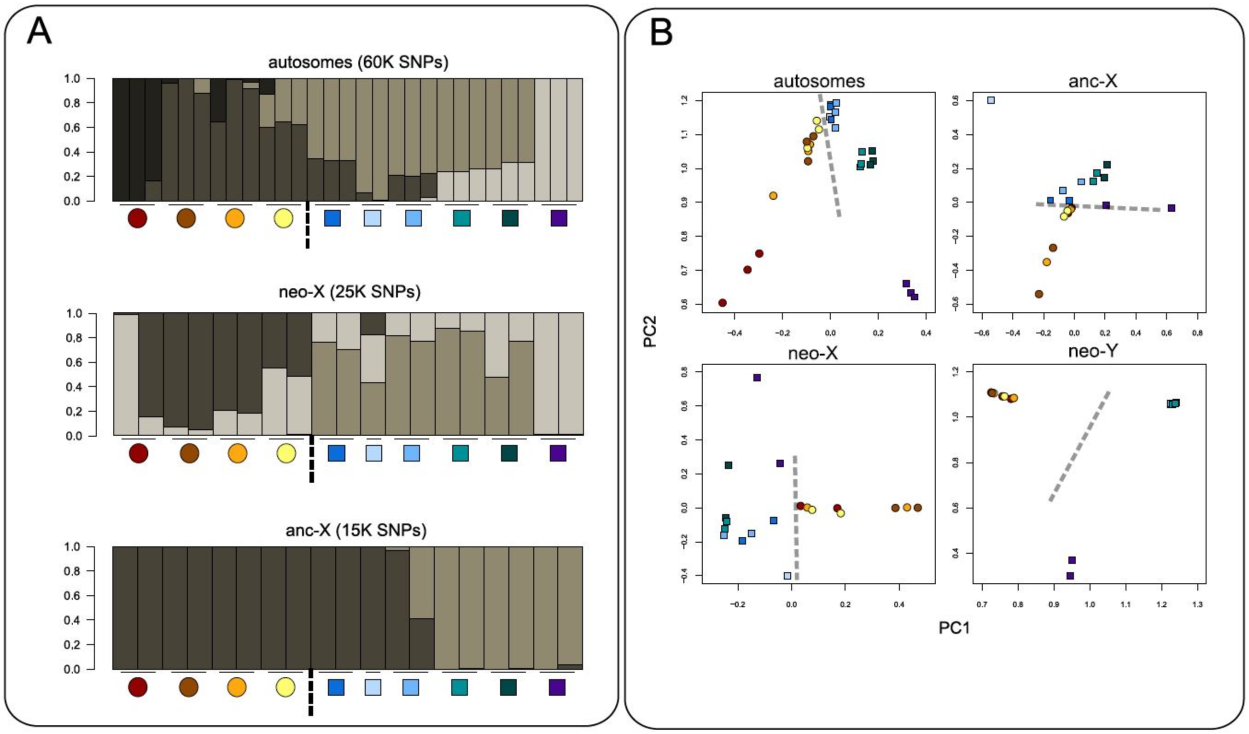

Supplementary Figure 7. Whole genome population genetic structure. (**A**) Results from STRUCTURE analysis showing the best *K* for autosomes (*K*=4), neo-X (*K*=3), and anc-X (*K*=2). (**B**) Principle component analyses for the autosomes, anc-X, neo-X and neo-Y SNPs. Population symbols correspond to Fig. 2a and the dashed line represents the hybrid male sterility boundary.

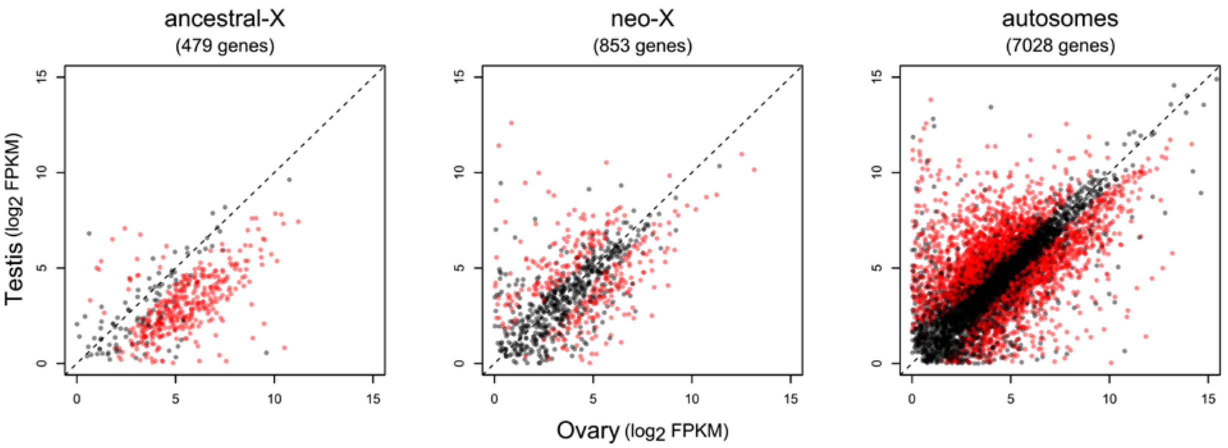

Supplementary Figure 8. Gene expression in testis and ovary. Expression profiles for genes found expressed in both the testis and ovary (FPKM ≥ 1) from three linkage categories (anc-X, neo-X and autosomes). Total number of expressed genes per linkage category is shown at the top of each plot. Each gene is represented as a point and differentially expressed genes are highlighted in red. The dashed line represents equivalent expression in both tissues.

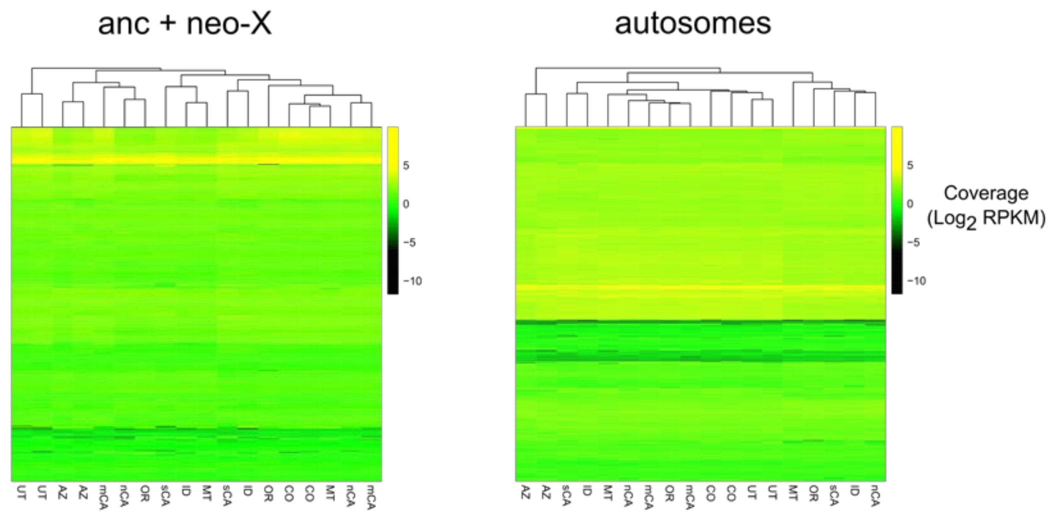

Supplementary Figure 9. Sequencing coverage of autosomal and X chromosome scaffolds. Clustered heatmaps of male coverage (Log_2 Reads Per Kilobase of sequence per Million mapped reads) of mountain pine beetle anc + neo-X and autosomal scaffolds. Each column represents a different beetle and each scaffold is shown as a different row. Population identifiers correspond to Fig. 2a.

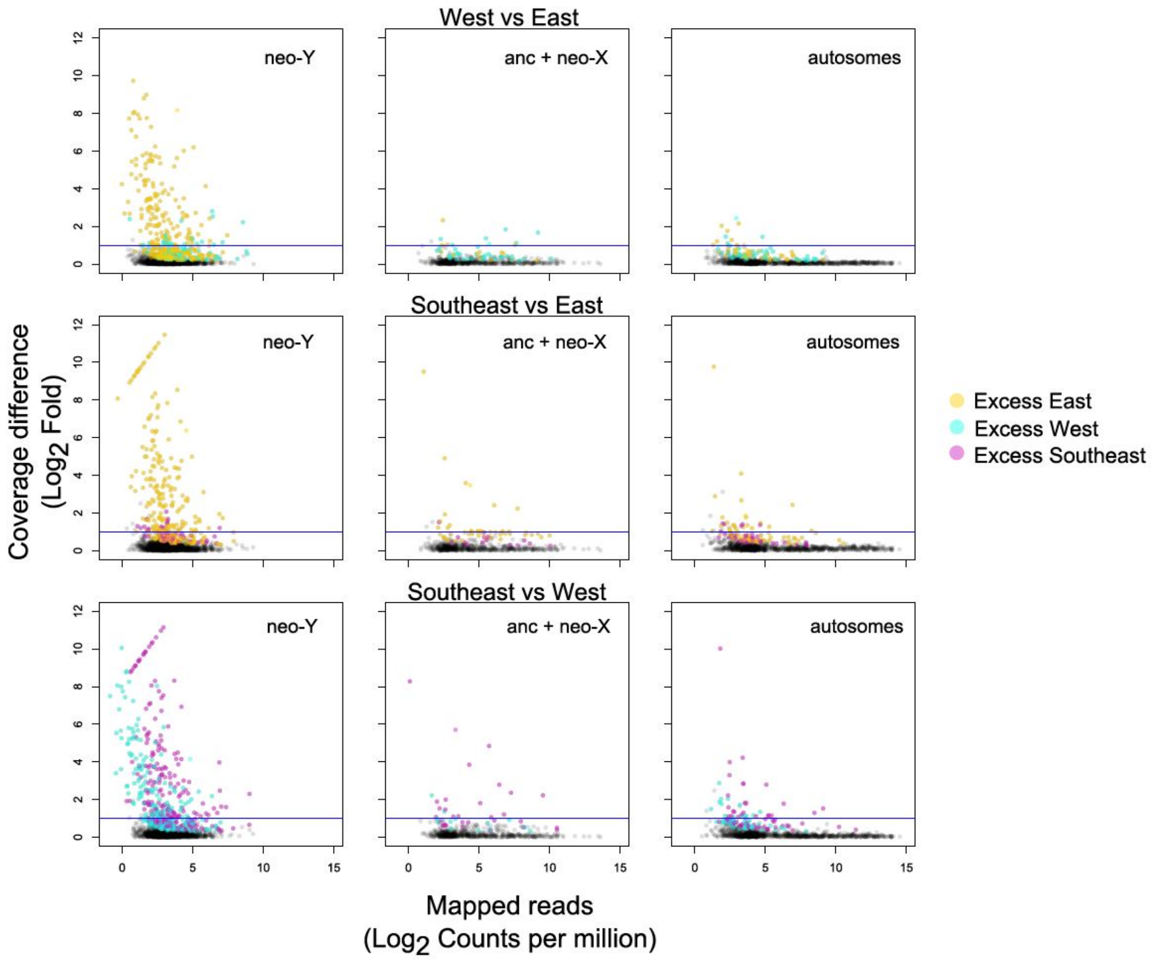

Supplementary Figure 10. Sequencing coverage differences in the mountain pine beetle partitioned by linkage category. Differences in sequencing coverage over scaffolds from the different linkage categories in pairwise comparisons between West, East and Southeast beetles. Each point is a different scaffold. Points are highlighted when found to have significant differences in sequencing coverage (based on edgeR analyses) and are colored to represent which beetles showed the excess in coverage (East, West, or Southeast). The blue line denotes a two-fold excess in coverage.

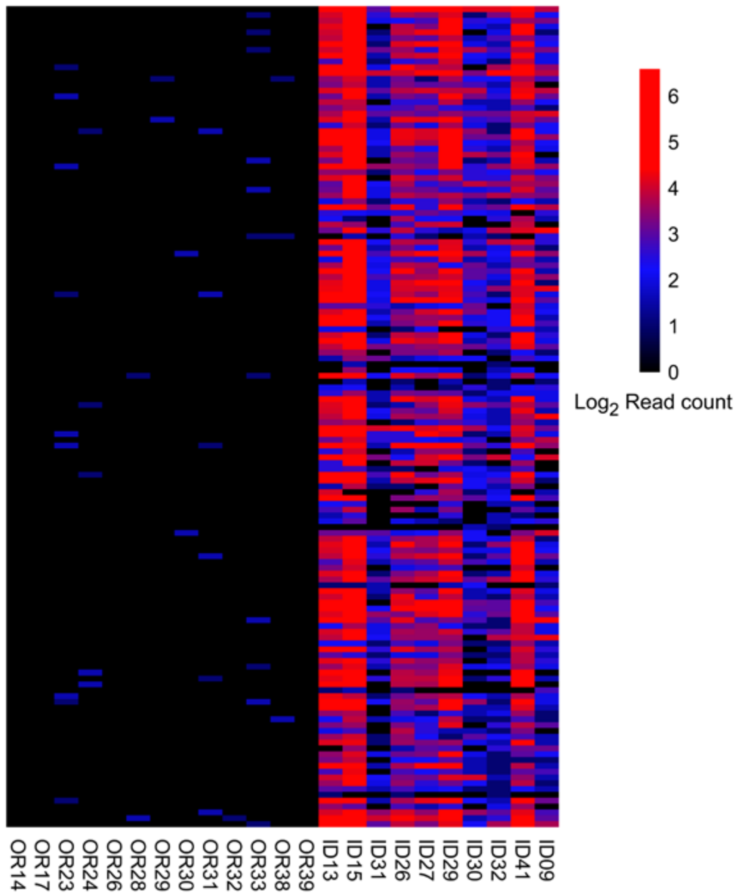

Supplementary Figure 11. Fixation of neo-Y deletions in the OR (West) population. OR (left, 13 individuals) and ID (right, 10 individuals) raw sequencing coverage of 141 RADtags located in neo-Y scaffolds identified as deleted in West beetles from whole genome sequencing analyses. Each row represents a RADtag (region adjacent to a restriction enzyme cut site in the genome). Slight differences in coverage among ID individuals was primarily due to differences in sequencing depth per individual and the stochastic nature of RADseq.

Supplementary Table 1. Results of crossing experiments testing for postzygotic reproductive isolation in MT × nCA and sCA × AZ. The MT × nCA and sCA × AZ crosses were analyzed separately and values followed by the same letter are not significantly different within a column (Tukey's HSD test). Total egg hatch and gallery length shown as means and SE. An individual was considered fertile if ≥ 1 egg hatched.

Population cross	Sex of hybrid	Backcross population	Number of bakcrosses	Proportion fertile	Total egg hatch	Gallery length (cm)
MT♀ x nCA♂	♀	nCA	17	1.00 (0.00)	101.18(10.02)c	41.06 (4.21)ab
		MT	15	1.00 (0.00)	83.87(9.39)c	39.07 (3.81)ab
	♂	nCA	16	0.31 (0.12)	27.56 (10.77)ab	35.34 (4.20)ab
		MT	16	0.13 (0.08)	7.94 (6.36)a	24.53 (3.41)a
nCA♀ x MT♂	♀	nCA	17	1.00 (0.00)	88.18 (9.00)c	43.70 (3.56)b
		MT	17	1.00 (0.00)	75.94 (10.64)c	35.50 (5.22)ab
	♂	nCA	16	1.00 (0.00)	74.69 (11.22)c	38.72 (4.20)ab
		MT	15	0.87 (0.09)	67.73 (11.03)bc	38.13 (4.36)ab
sCA ♀ x AZ ♂	♀	AZ	24	0.96 (0.04)	65.79 (6.39)bc	41.97 (2.42)bc
		sCA	31	0.97 (0.03)	68.13 (5.42)c	46.09 (2.68)c
	♂	AZ	20	0.05 (0.05)	3.25 (3.25)a	28.64 (2.67)a
		sCA	26	0.00 (0.00)	0.00 (0.00)a	31.76 (2.36)ab
AZ ♀ x sCA ♂	♀	AZ	24	0.92 (0.06)	41.46 (5.82)b	35.47 (2.33)ac
		sCA	24	0.96 (0.04)	53.83 (6.28)bc	37.93 (3.05)ac
	♂	AZ	9	0.00 (0.00)	0.00 (0.00)a	31.94 (3.17)ac
		sCA	25	0.00 (0.00)	0.00 (0.00)a	30.94 (2.34)a
AZ ♀ x AZ ♂	*	*	24	1.00 (0.00)	59.96 (5.20)bc	37.38 (2.35)ac
sCA ♀ x sCA ♂	*	*	28	0.96 (0.04)	57.29 (5.23)bc	42.18 (2.11)bc

Supplementary Table 2. Autosomal Weir and Cockerham's weighted F_{st} (below diagonal), D_{xy} (above diagonal) and π (diagonal) for all populations (excluding CAN).

**sCA**	0.0065	0.0076	0.0076	0.0078	0.0080	0.0080	0.0079	0.0083	0.0083
**mCA**	0.190	0.0066	0.0069	0.0069	0.0068	0.0068	0.0073	0.0074	0.0075
**nCA**	0.136	0.002	0.0072	0.0070	0.0070	0.0070	0.0073	0.0074	0.0078
**OR**	0.192	0.028	0.013	0.0068	0.0066	0.0066	0.0070	0.0070	0.0076
**ID**	0.259	0.064	0.060	0.012	0.0062	0.0062	0.0066	0.0066	0.0074
**MT**	0.262	0.074	0.071	0.026	0.001	0.0062	0.0065	0.0065	0.0073
**UT**	0.281	0.127	0.135	0.110	0.104	0.089	0.0058	0.0062	0.0065
**CO**	0.317	0.154	0.148	0.116	0.096	0.088	0.069	0.0058	0.0065
**AZ**	0.452	0.338	0.341	0.342	0.363	0.353	0.286	0.286	0.0042
	**sCA**	**mCA**	**nCA**	**OR**	**ID**	**MT**	**UT**	**CO**	**AZ**

Supplementary Table 3. Results of tests for admixture using the three-population test (f_3 statistic). Shown are all tests were the target population draws highly significant signatures of admixture from the two source populations (z-score < -10).

Target	Source 1	Source 2	f_3 statistic	SE	z-score
OR	MT	nCA	-0.004	0.000119	-33.672
OR	nCA	ID	-0.00418	0.00013	-32.097
OR	CO	nCA	-0.00343	0.000127	-26.973
OR	mCA	MT	-0.00312	0.00013	-24.078
OR	MT	sCA	-0.0059	0.000254	-23.278
OR	ID	sCA	-0.00693	0.000305	-22.709
OR	mCA	ID	-0.00349	0.000164	-21.268
OR	CO	sCA	-0.0048	0.000251	-19.144
OR	CAN	nCA	-0.0035	0.000193	-18.106
OR	mCA	CO	-0.0025	0.00014	-17.817
OR	UT	nCA	-0.00248	0.000172	-14.400
nCA	mCA	OR	-0.0033	0.000124	-26.756
nCA	MT	sCA	-0.00777	0.000345	-22.536
nCA	CO	sCA	-0.00723	0.000333	-21.698
nCA	sCA	OR	-0.00586	0.000273	-21.489
nCA	ID	sCA	-0.00861	0.000404	-21.322
nCA	mCA	sCA	-0.00532	0.000304	-17.529
nCA	mCA	MT	-0.00243	0.000205	-11.859
nCA	mCA	ID	-0.00261	0.000222	-11.731
nCA	mCA	CO	-0.00238	0.000212	-11.223
MT	CO	OR	-0.00235	0.000124	-18.908
MT	CO	ID	-0.00214	0.000117	-18.223
MT	CAN	CO	-0.00292	0.000164	-17.822
MT	UT	ID	-0.00285	0.000188	-15.123
MT	CO	nCA	-0.00179	0.000132	-13.564
MT	mCA	CO	-0.00173	0.000128	-13.510
MT	ID	AZ	-0.00288	0.000225	-12.811
MT	UT	OR	-0.00198	0.000157	-12.616
MT	CAN	ID	-0.00229	0.000228	-10.032
mCA	UT	nCA	-0.00237	0.000147	-16.157
mCA	nCA	AZ	-0.00254	0.000169	-15.051
mCA	CAN	nCA	-0.00215	0.000154	-13.977
mCA	ID	sCA	-0.00407	0.000382	-10.655
mCA	MT	sCA	-0.0034	0.000335	-10.168
ID	MT	OR	-0.00178	0.000096	-18.576
ID	CO	OR	-0.002	0.000124	-16.090
ID	MT	nCA	-0.0016	0.000113	-14.184
ID	mCA	MT	-0.00142	0.000137	-10.371
UT	CAN	AZ	-0.00227	0.000205	-11.080
UT	MT	AZ	-0.0027	0.00025	-10.795
UT	ID	AZ	-0.00273	0.000271	-10.071
CO	MT	AZ	-0.00293	0.000181	-16.173
CO	ID	AZ	-0.00368	0.000263	-14.001

Supplementary Table 4. Results from D-statistic tests across different genomic partitions. A positive D-statistic (i.e., an excess of ABBA sites) indicates gene flow between P2 and P3 given the relationship (((P1, P2), P3), O). Z-scores > 3 were considered significant (see Methods).

Partition	Comparison	ABBA	BABA	BBAA	D-statistic	SE	Z-score
neo-Y	(((CO, ID), OR), JPB)	1.8	2.8	138.5	-0.222	0.20546	-1.1
	(((sCA, OR), ID), JPB)	3.5	2.8	179.8	0.120	0.14223	0.8
anc-X	(((CO, ID), OR), JPB)	3,346.1	2,096.6	3,131.9	0.230	0.00481	47.8
	(((sCA, OR), ID), JPB)	2,936.1	2,215.4	3,074.6	0.140	0.00444	31.5
neo-X	(((CO, ID), OR), JPB)	2,337.1	1,650.4	4,489.6	0.172	0.00890	19.4
	(((sCA, OR), ID), JPB)	3,525.5	1,859.5	4,574.8	0.309	0.00469	66.0
Autosomes	(((CO, ID), OR), JPB)	26,499.5	20,591.3	26,583.3	0.125	0.00509	24.7
	(((sCA, OR), ID), JPB)	38,864.4	19,977.4	23,677.4	0.321	0.01505	21.3

Supplementary Table 5. Results from enrichments tests. Genes (observed(expected)) were considered tissue specific when a gene was expressed in the focal tissue at FPKM > 1 (top), or FPKM > 10 (bottom) and not expressed in other tissues (FPKM = 0).

FPKM > 1 IN FOCAL TISSUE

	Testis	Ovary	Male head	Female head (rep 1)	Female head (rep 2)
neo-Y	344(144)		26(4)		
autosomes	1607(1723)	646(671)	23(42)	26(29)	73(79)
anc-X	12(113)	77(44)	1(3)	3(2)	9(5)
neo-X	274(257)	128(100)	5(6)	6(4)	18(12)

not signficant
enriched
depleted

observed(expected)

FPKM > 10 IN FOCAL TISSUE

	Testis	Ovary	Male head	Female head (rep 1)	Female head (rep 2)
neo-Y	74(133)		10(1)		
autosomes	385(394)	211(224)	8(16)	7(7)	18(19)
anc-X	3(26)	24(15)	1(1)	0(0)	2(1)
neo-X	49(59)	43(33)	2(2)	2(1)	5(3)

Supplementary Table 6. Neo-Y gene intervals identified as deleted in West and Southeast beetles. Shown are whole genome sequencing mean read counts and standard error over gene intervals for East, West, and Southeast males. Gene expression (FPKM) in East beetles shown for female heads (2 replicates), ovary, testis and male head. Genes with male biased patterns of expression (FPKM > 1 in male head or testis and FPKM < 1 in female head and/or ovary) in bold.

Gene interval	Gene ID	Status	Mean East read count	SE	Mean West read count	SE	Mean Southeast read count	SE	Female head(1) FPKM	Female head(2) FPKM	Ovary FPKM	Testis FPKM	Male head FPKM
**Seq_1096932:1934-3303**	**YQE_00266**	private West deletion	196.6	31.1	0.0	0.0	195.5	43.5	0.0	0.0	0.0	2.2	1.7
Seq_1097260:1821-2681	YQE_00324	private West deletion	107.3	15.4	0.0	0.0	72.5	30.5	0.9	1.1	0.3	1.0	0.6
**Seq_1100163:390-1064**	**YQE_01039**	private West deletion	86.8	8.3	0.0	0.0	70.0	29.0	0.2	0.4	0.2	6.7	6.7
Seq_1102651:14497-14766	YQE_05104	private West deletion	20.0	2.6	0.0	0.0	15.0	6.0	0.0	0.0	0.0	0.0	0.0
Seq_1102701:11352-11630	YQE_06450	private West deletion	50.4	2.8	0.0	0.0	43.5	3.5	0.0	0.0	0.0	0.0	0.0
**Seq_1102857:11881-12625**	**YQE_09570**	private West deletion	113.8	9.3	0.0	0.0	104.5	22.5	0	0	0.0	2.9	0.0
**Seq_1102356:1024-9163**	**YQE_03557**	private West deletion	245.3	35.2	3.8	1.0	163.5	36.5	0.0	0.1	0.1	12.5	0.5
**Seq_1101211:3136-3483**	**YQE_01382**	private West deletion	21.4	1.7	0.5	0.3	26.0	7.0	0.1	0.0	0.0	3.5	1.2
Seq_1102622:22124-24200	YQE_05005	private West deletion	85.5	6.7	2.0	0.4	65.0	26.0	0.0	0.0	0.0	0.1	0.2
Seq_1102189:4856-7492	YQE_02942	private West deletion	323.4	26.1	10.1	2.2	232.5	80.5	1.6	1.0	1.1	1.4	2.2
Seq_1096815:25-617	YQE_00241	private West deletion	90.6	6.4	3.8	1.2	81.0	25.0	0.0	0.1	0.1	0.1	0.0
Seq_1099468:921-3356	YQE_00714	private Southeast deletion	184.5	15.4	139.6	20.7	0.0	0.0	3.6	5.2	3.2	2.1	3.2
**Seq_1100692:3-1334**	**YQE_01207**	private Southeast deletion	128.5	9.0	98.3	8.1	0.0	0.0	0.3	0.2	0.9	2.3	3.2
**Seq_1100519:201-1682**	**YQE_01153**	private Southeast deletion	94.8	10.1	81.9	14.6	0.0	0.0	0.0	0.1	0.0	0.9	1.1
**Seq_1099365:551-4330**	**YQE_00678**	private Southeast deletion	460.9	13.8	379.5	39.2	15.5	0.5	0.0	0.0	0.0	1.7	0.4
Seq_1102380:29420-30470	YQE_03635	private Southeast deletion	169.3	19.1	118.0	21.3	0.0	0.0	0.0	0.1	0.0	0.3	0.9
Seq_1101865:16841-17348	YQE_02271	private Southeast deletion	66.9	3.5	58.1	5.4	0.0	0.0	1.1	1.3	1.5	0.3	2.4
Seq_1102226:13-1150	YQE_03004	shared deletion	86.8	7.8	0.0	0.0	0.0	0.0	0.0	0.0	0.0	0.4	0.0
**Seq_1102822:8620-9099**	**YQE_09046**	shared deletion	57.0	1.9	0.0	0.0	0.0	0.0	0.0	0.0	0.0	4.1	0.0
Seq_1099628:681-1283	YQE_00813	shared deletion	50.6	4.0	0.5	0.4	0.0	0.0	0.0	0.0	0.0	0.0	0.0
Seq_1102226:2229-8652	YQE_03005	shared deletion	289.5	13.8	13.4	3.5	3.0	2.0	0.0	0.3	0.0	0.0	0.0

Supplementary References

1. Bracewell, R. R., Pfrender, M. E., Mock, K. E. & Bentz, B. J. Cryptic postzygotic isolation in an eruptive species of bark beetle (*Dendroctonus ponderosae*). *Evolution* **65**, 961-975 (2011).
2. Hay, J.C. Experimental crossing of mountain pine beetle with the black hills beetle. Ann. Entomol. Soc. Am. **49**, 567-571 (1956).
3. Bentz, B. J., Bracewell, R. R., Mock, K. E. & Pfrender, M. E. Genetic architecture and phenotypic plasticity of thermally-regulated traits in an eruptive species, *Dendroctonus ponderosae*. *Evol. Ecol.* **25**, 1269-1288 (2011).
